# Vitamin D intake and gastric cancer in Viet Nam: a case-control study

**DOI:** 10.1186/s12885-022-09933-2

**Published:** 2022-08-01

**Authors:** Minh Thien Nguyen, Nhi Ngoc Yen Huynh, Dai Duc Nguyen, Nguyen Ha Ta, Tai Van Nguyen, Huy Thanh Dang, Ngoan Tran Le

**Affiliations:** 1 School of Medicine, International University of Health and Welfare, Narita City, Japan; 2 Institute of Research and Development, Duy Tan University, Da Nang City, Viet Nam; 3 Department of Public Health, School of Medicine, International University of Health and Welfare, Narita City, Japan

**Keywords:** Vitamin D, Gastric cancer, Epidemiology, Case-control study, Viet Nam

## Abstract

**Background:**

Most recent laboratory studies have suggested a promising role of vitamin D and its analogs as novel chemotherapeutic agents for cancer treatment. However, epidemiological evidence, especially regarding the effects of vitamin D on gastric cancer is still inconsistent.

**Objectives:**

Our research aimed to evaluate the associations between vitamin D intake and the risk of developing gastric cancer through a case-control study in North Vietnam.

**Methods:**

We accessed databases of the previous completed case-control studies to derive 1182 incident gastric cancer cases and 2995 hospital controls selected from hospitals in Hanoi from 2003 to 2019. Vitamin D intake was computed by multiplying the food frequency intake with nutrient content based on the Viet Nam Food Composition Tables. Data were collected through face-to-face interviews by trained interviewers using the validated semi-quantitative food frequency and demographic lifestyle questionnaires. The odds ratio and 95% confidence interval (OR and 95%CI) were estimated using unconditional logistic regression analysis.

**Results:**

We observed a continual decline in gastric cancer risk according to the level-up of vitamin D intake in both genders, men, and women [Fifth vs. bottom quintile, OR, 95%CI: 0.68 (0.53, 0.86), OR, 95%CI: 0.72 (0.53, 0.97), OR, 95%CI: 0.58 (0.38, 0.89), respectively. Per increment quintile, the statistically significant decreased risk was seen by 7% in men and 13% in women. The significant inverse association between vitamin D intake remained in the subgroups of ever and never tobacco smoking; negative and positive *H. pylori* infection.

**Conclusion:**

The findings suggested that sufficient vitamin D intake was associated with a lower risk of Gastric Cancer in the Vietnamese population.

## Introduction

Over the last decades, the overall incidence of Gastric cancer (GC) has been declining, attributed to the eradication of *Helicobacter Pylori* (*H. pylori*) and the use of refrigeration to preserve foods [1]. However, GC is still the fifth most common cancer worldwide and the third most common cause of death from cancer responsible for over 1,000,000 new cases and 783,000 deaths in 2018 [2].

The highest GC incidence was recorded in Asian countries: 1st: South Korea, 2nd: Mongolia, 3rd: Japan, 4th: China, which accounts for more than half of all cases. Viet Nam ranks 10th with 15.9 new cases per 100,000 [2]. A study on non-cardia GC conducted in the two biggest cities of Viet Nam showed a male predominance and a strong relationship with *H. pylori* infection [3].

GC results from a combination of lifestyle, environmental factors, and the accumulation of specific genetic alterations [4]. GC can be divided into 2 topographical subtypes: cardia and non-cardia GC. The two subtypes share common risk factors including older age, male sex, smoking, radiation, and family history [5]. Non-cardia GC has been steadily declining globally and is primarily associated with *H. pylori* infection, low socioeconomic status, low consumption of fruits and vegetables, and high salt intake. On the other hand, cardia-GC has been increasing particularly in high-income countries and is mainly linked to obesity and Gastroesophageal reflux disease [2, 5].

Vitamin D comprises a group of fat-soluble sitosterol hormones with two major forms of vitamin D2 and vitamin D3. D3 is made in the skin under UV light, whereas D2 is derived from the plant sterol ergosterol [6]. Both forms are metabolized first to 25 hydroxyvitamin D (25OHD or Calcidiol) in the liver, then to the biologically active hormonal form 1,25-dihydroxy vitamin D (1,25(OH)2D or Calcitriol) mainly by the kidney [6, 7]. Calcitriol is the ligand for the vitamin D receptor (VDR), a transcription factor, binding to sites in the DNA called vitamin D response elements (VDREs) [7]. Recent research found that VDRs are widely distributed throughout the body even in tissues not involved with calcium and phosphate homeostasis, while VDREs are also present in a large number of human genes involved in a wide range of classical and non-classical roles, in a cell-specific fashion [6–8]. The increasing evidence from studies in cell culture and experimental models suggests that vitamin D has many roles beyond calcium homeostasis that regulate multiple cellular processes such as inhibiting differentiation, metastasis, proliferation, and inducing apoptosis and cell cycle arrest [9]. These proposed mechanisms support vitamin D’s role as a potent chemotherapy agent, therefore, the interest in vitamin D and research on its suggested anti-cancer effects has greatly increased over the last decade. Our study aimed to evaluate the correlation between dietary intake of vitamin D and GC in the Vietnamese population.

## Materials and methods

### Study population

We derived data from databases of the previous completed case-control studies performed in the hospital located in Hanoi from 2003 to 2019. Study participants included 1182 incident gastric cancer cases and 2995 hospital controls. These previous completed case-control studies were supported by the Vietnam Ministry of Science and Technology and the investigators followed the national guidelines and regulations. The designed research protocols were approved by the scientific committees of the Hanoi Medical University [10], the Institutional Review Board of Hanoi Medical University [11], and the Ministry of Science and Technology [12, 13].

The study model consists of 1182 cases and 2995 controls selected from in-patients in hospitals, in Hanoi, Viet Nam. The cases are patients diagnosed as having GC by histopathological evaluation and confirmation. Controls included non-cancer patients in the same department during the same period. The study methods have been described and published elsewhere [11, 14].

### Recruitment of cases

Gastric cancer cases were recruited from the list of patients who have been admitted to the Bach Mai hospital, Viet Duc Hospital, National Cancer hospital, and Hanoi Medical University hospital for surgical treatment to remove gastric cancer tumors for the first time. Every day, patients have entered the inpatient wards for their surgical treatment the next day. The eligible cancer patients were invited to participate in the present study and investigators have to get their voluntary agreement before interviews, face-to-face, bedside by the trained interviewers to collect data [10–14].

### Recruitment of hospital controls

The hospital control cases were also weekly recruited from the same list of patients who have been admitted to the Bach Mai hospital for surgical treatment for non-cancer diseases. The Bach Mai hospital is located nearby the other three health facilities of Viet Duc hospital, the National cancer hospital, and Hanoi Medical University hospital where cases were recruited. These control cases were daily also checked-in at the Bach Mai hospital from 1 PM to 5 PM, patients also entered the inpatient wards for their surgical treatment on the next day. The eligible hospital controls were invited to participate in the present study and researchers have to get their voluntary agreement before interviews, face-to-face, bedside by the trained interviewers to collect data [10–14].

### Exposure estimation

The designed Demographics & Lifestyles, Semi-Quantitative Food Frequency Questionnaires (SQFFQ) were used to collect data from all participants. SQFFQ is a validated and reproducible set of 192 questions that reflect detailed dietary habits as well as other elements that have an impact on one’s health status over 1 year. Among those, 123 questions were related to usual food items, along with sub-questions providing information about the amount, frequency, and methods of cooking of each item. Participants were interviewed by trained interviewers on the day immediately before their surgical treatment [10–14]. The nutrient composition of food intake was based on the Viet Nam Food Composition Tables [15]. From these data sources, we calculated the estimated total intake of each nutrient including vitamin D intake for each study participant in the present study.

### Validation of the data collection tool

The SQFFQ was validated by comparing nutrient intake by two methods of 24-hour food records for three consecutive weekdays (The reference group) and the designed SQFFQ by 310 eligible study participants. The SQFFQ was completed one time during autumn 2018. The designed SQFFQ was being validated as having good characteristics of feasibility, practical, and reliability in the general populations located in North Viet Nam based on the estimated indicators of a small correlation for lipid (adjusted R^2^ = 0.20); a moderate correlation for protein (adjusted R^2^ = 0.38) and carbohydrate (adjusted R^2^ = 0.36), a strong correlation for energy intake (adjusted R^2^ = 0.53) between two methods of data collection [16].

### A routine determination of *H. pylori* infection

The previous completed case-control studies have determined the status of *H. pylori* infection based on a routine laboratory test before performing surgical procedures of treatment for each case and control. Anti-*H. pylori* serum IgG antibody titers were tested using an enzyme-linked immunosorbent assay (ELISA), based on the sandwich principle, through the use of an *H. pylori* IgG ELISA kit (RE56381; IBL International, Hamburg, Germany). *H. pylori* status was classified into three groups, based on a cut-off index (COI) provided by the manufacturer: negative (COI < 0.8), equivocal (0.8–1.2), and positive (> 1.2) for the blood samples collected during 2018-2019. From 2003 to 2011, Anti-*H. pylori* serum IgG antibody titers were examined using the *Pyloriset* EIA-G III, Orion Diagnostica to perform the IgG ELISA test. The cut-off level of < 20 U/ml was considered a negative *H. pylori* status.

### Statistical analysis

Odds ratio and 95% confidence interval (OR and 95%CI) were estimated using unconditional logistic regression analysis adjusted for age, education, smoking, BMI, family history of cancer, blood group (A, AB, B, O), alcohol drinking, fridge available at home, four periods of data collection, energy intake, and serum status of *H. pylori* infection. The OR and 95%CI were estimated for the entire study population, as well as the subgroup of ever and never tobacco smoking; negative and positive *H. pylori* infection.

### Ethical approval

#### Ethics approval and consent to participate

The authors confirm to follow the study protocol that was approved by the ethics committees of the IRB-International University of Health and Welfare, Japan (Approval number 19-Ig-17) dated 27 May 2019. This study uses the database of existing previous completed case-control studies. Participants were anonymous and personal information was coded which cannot identify participants. Each participant was coded with an ID. Data is saved into a USB stick memory with a password.

All methods were performed and carried out by relevant ethical guidelines and Vietnam’s national regulations. The existing previous completed case-control studies were performed in the hospitals located in Hanoi from 2003 to 2019. The study protocol was approved by the ethics committees of Ha Noi Medical University, number 3918/HMUIRB dated 25 December 2018. We obtained verbal informed consent from all participants. Such as, for the week from 8 to 12 Jan. 2018, among the list of 81 patients admitted and were planning to be treated by surgeries at the Dept. of Surgery of Bach Mai hospital, we invited 34 of 81 patients who agreed to participate in the study as voluntary. Study participants have completed a questionnaire survey and allowed investigators access to data of routine laboratory tests of *H. pylori* infection and hepatitis viral infection. According to the guideline for epidemiological studies in Vietnam and Japan in 2002, written informed consent was not required for an observational study based on a questionnaire survey. Completion and return of the questionnaire were considered implied consent.

## Results

By comparison between quintiles, there was a higher intake of lipid (mean 45.8 g versus 26.4 g), protein (mean 90.2 g versus 59.9 g), and energy (1806.7 Kcal versus 1638.4 Kcal) in the 5th quintile than in the 1st quintile. In contrast, carbohydrate intake was lower in the 5th quintile than in the 1st quintile (261.0 g versus 193.3 g), Table 1.

**Table 1:** Characteristics of demographic and macronutrients intake of quintile 1 and quintile 5

	Quintile 1			Quantiles			Quintile 5			Quantiles		
Variable	n	Mean	S.D.	Min	Median	Max	n	Mean	S.D.	Min	Median	Max
**Age**	837	56.3	11.6	20.0	57.0	85.0	835	54.1	12.5	15.0	56.0	86.0
**BMI**	795	20.1	3.1	13.3	19.7	31.2	825	21.4	3.0	12.1	21.3	37.8
**Energy (Kcal)**	837	1638.4	415.5	414.0	1655.0	2992.0	835	1806.7	473.8	785.0	1763.0	4361.0
**Protein (g)**	837	59.9	17.5	19.3	58.6	148.0	835	90.2	27.7	37.4	86.1	266.5
**Lipid (g)**	837	26.4	12.6	3.1	24.0	78.3	835	45.8	19.3	11.5	41.5	172.9
**Carbohydrate (g)**	837	293.3	84.2	67.5	296.8	516.3	835	261.0	85.5	87.3	239.8	663.6
**Fiber (g)**	837	5.5	2.3	1.1	5.1	17.0	835	6.4	2.9	1.9	5.7	24.5

The mean dietary vitamin D intake in the highest quintile was 11.9 μg/day, and that of the lowest quintile was 0.4 μg/day. A continual decline in cancer risk according to the increment quintile of vitamin D intake was recorded (OR and 95%CI: 0.84 (0.80-0.88), *p* = 0.001). After adjusting for age, education, smoking, BMI, family history of cancer, blood group (A, AB, B, O), alcohol drinking, fridge at home, four periods of data collection, energy intake, and serum status of *H. pylori* infection, a steady decline in adjusted ORs from each level up of vitamin D intake quintile was also observed (adjusted OR and 95%CI: 0.91 (0.86-0.96), *p* = 0.000). A statistically significant decreased risk of GC was found at the highest 5th quintile compared to the lowest quintile (adjusted OR and 95%CI, 5th versus 1st quintiles: 0.68 (0.53-0.86), *p* = 0.002), Table 2.

**Table 2:** Vitamin D intake and gastric cancer, men and women combined

Mean (μg/day)	Control	Cancer	Total	Crude OR (95% CI)	p	Adjusted OR (95% CI)^a^	p
0.4	553	284	837	1.00 (reference)		1.00 (reference)	
1.9	550	284	834	1.01 (0.82, 1.23)	0.958	1.13 (0.91, 1.40)	0.271
3.6	614	229	843	0.73 (0.59, 0.89)	0.003	0.88 (0.71, 1.11)	0.284
5.6	608	220	828	0.70 (0.57, 0.87)	0.001	0.91 (0.72, 1.14)	0.404
11.8	670	165	835	0.48 (0.38, 0.60)	0.000	0.68 (0.53, 0.86)	0.002
	per increment quintile			0.84 (0.80, 0.88)	0.000	0.91 (0.86, 0.96)	0.000
Total	2995	1182	4177				

^a^Adjusted for sex, age, education, smoking, BMI, family history of cancer, blood group (A, AB, B, O), alcohol drinking, fridge at home, four periods of data collection, energy intake, and serum status of *H. pylori* infection

Stratified analysis according to sex-specific quintile also reached similar outcomes in both men and women subgroups (adjusted OR and 95%CI, 5th versus 1st quintiles: 0.72 (0.53-0.97), *p* = 0.028; and adjusted OR and 95%CI, 5th versus 1st quintiles: 0.58 (0.38, 0.89), *p* = 0.013, respectively). Again, the continual decline in cancer risk according to the increment quintile of vitamin D intake was recorded (adjusted OR and 95%CI: 0.93 (0.87, 0.99), *p* = 0.027 for men and adjusted OR and 95%CI: 0.87 (0.80, 0.96, *p* = 0.003 for women). Tables 3 and 4.

**Table 3:** Vitamin D intake and gastric cancer in men

Mean (μg/day)	Control	Cancer	Total	Crude OR (95% CI)	p	Adjusted OR (95% CI)^a^	p
0.4	287	184	471	1.00 (reference)		1.00 (reference)	
1.9	329	186	515	0.88 (0.68, 1.14)	0.339	0.99 (0.76, 1.30)	0.942
3.6	381	167	548	0.68 (0.53, 0.89)	0.004	0.85 (0.64, 1.12)	0.242
5.5	379	163	542	0.67 (0.52, 0.87)	0.003	0.90 (0.68, 1.18)	0.440
12.0	381	123	504	0.50 (0.38, 0.66)	0.000	0.72 (0.53, 0.97)	0.028
	per increment quintile			0.85 (0.80, 0.90)	0.000	0.93 (0.87, 0.99)	0.027
Total	1757	823	2580				

^a^Adjusted for age, education, smoking, BMI, family history of cancer, blood group (A, AB, B, O), alcohol drinking, fridge at home, four periods of data collection, energy intake, and serum status of *H. pylori* infection

**Table 4:** Vitamin D intake and gastric cancer in women

Mean (μg/day)	Control	Cancer	Total	Crude OR (95% CI)	p	Adjusted OR (95% CI)^a^	p
0.4	266	100	366	1.00 (reference)		1.00 (reference)	
1.9	221	98	319	1.18 (0.85, 1.64)	0.328	1.42 (1.00, 2.01)	0.050
3.6	233	62	295	0.71 (0.49, 1.02)	0.062	0.96 (0.65, 1.41)	0.818
5.6	229	57	286	0.66 (0.46, 0.96)	0.029	0.91 (0.61, 1.35)	0.644
11.5	289	42	331	0.39 (0.26, 0.57)	0.000	0.58 (0.38, 0.89)	0.013
	per increment quintile			0.79 (0.73, 0.86)	0.000	0.87 (0.80, 0.96)	0.003
Total	1238	359	1597				

^a^Adjusted for age, education, smoking, BMI, family history of cancer, blood group (A, AB, B, O), alcohol drinking, fridge at home, four periods of data collection, energy intake, and serum status of *H. pylori* infection

The significant inverse association between vitamin D intake remained in the subgroups of ever and never tobacco smoking; negative and positive *H. pylori* infection. An adjusted OR and 95%CI, 5th versus 1st quintiles: 0.41 (0.29, 0.60), *p* = 0.000 for never smoker; OR and 95%CI: 0.64 (0.46, 0.94), *p* = 0.012 for ever smoker; OR and 95%CI: 0.23 (0.13, 0.39), *p* = 0.000 for negative *H. pylori* infection; OR and 95%CI: 0.50 (0.33, 0.74, *p* = 0.001 for positive *H. pylori* infection (data not shown).

## Discussions

We observed strong beneficial protection of vitamin D intake against gastric cancer in the entire study population in general and in both men and women in particular. Intake of vitamin D of the estimated amount of 11 μg/day or higher (the 5th quintile) reduced about 32% of cancer incidence, which was 28% in men and 42% in women. The beneficial effect of vitamin D intake was consistent among the entire study population with each subgroup ever and never tobacco smoking; negative and positive *H. pylori* infection. The findings suggested that the inverse association appeared stronger in never smoker and negative *H. pylori* infection groups than in ever smoker and positive *H. pylori* infection groups. In addition, following the recommendation of the Institute of Health (US) of a daily dietary vitamin D intake of 400-600 IU (10-15 μg) for children to adults [17], the proportion of vitamin D deficiency was about 77.6% of 2995 persons in the control group. The findings suggested that vitamin D is possibly more protective in reducing the risk of gastric cancer in women when compared to men. The mechanisms of this effect are unclear. However, Estrogen hormone in women may have a protective role in the pathogenic pathway of GC or interact with the biological activity of vitamin D or its precursors. Besides, Vietnamese men are likely to have higher exposure to tobacco smoking and alcohol drinking than women [18]. These two established risks of gastric cancer might be a modification of the association between vitamin D and gastric cancer in the present study.

The present study has many strengthening points that include a relatively large sample size of 4177 study participants, in which 1182 cancer cases and 2995 hospital controls. The database has available variables for the adjustment that were included the infectious agent of *H. pylori* status, lifestyles factors of tobacco smoking and alcohol drinking; the social-economic status of education, and fridge at home; the host factor of blood groups, sex, age, and family history of cancer; and other factors of body mass index and energy intake. These advantage indicators will allow us to look at the association between vitamin D intake and the risk of gastric cancer.

Vitamin D can be obtained from sun exposure, supplements, and limited naturally occurring food, including oily fish, fish liver oil, mushroom, and egg yolk [19]. Besides, dermal synthesis remains the major route, accounting for 90% of vitamin D replenishment [20]. Most foods except for fatty fish contain little vitamin D unless fortified [6]. The vitamin D in fish is D3, whereas that used for fortification is often D2 (ergocalciferol), which is produced by UVB irradiation of the ergosterol in plants and mushrooms [7]. Both D3 and D2 are synthesized commercially and found in dietary supplements or fortified foods. After vitamin D was recognized as important for the prevention of rickets in the 1920s, in the US and Canada, vitamin-D-fortification in some foods like fluid milk, margarine, yogurts, plant-based beverages, cheeses, and juices was initiated voluntarily [20]. Owing to its fat-soluble nature, dietary vitamin D is absorbed with other dietary fats in the small intestine, therefore, the efficient absorption of vitamin D is dependent upon the presence of fat in the lumen [6, 21].

Vitamin D deficiency (VDD) is now considered a worldwide health issue, not only because of its consequence in bone diseases, but also its recently discovered association with the risk of cancers, autoimmune disorders, hypertension, and infectious disease [22]. VDD is diagnosed when measured serum 25OHD < 20 ng/mL [23]. VDD has become an emerging problem in Viet Nam as the reported prevalence of VDD was 30% in females and 16% in males [24]. The Institute of Health (US) recommended a daily dietary vitamin D intake of 400 IU (10 μg) for infants, 400-600 IU (10-15 μg) for children to adults, and 400-800 IU (10-20 μg) for elderly aged over 70 [17]. These amounts of intake were made concerning bone issues in the community thus they have limited value in the interest of cancer prevention. There haven’t been evidence-based scientific studies to determine the recommended vitamin D requirement for the Vietnamese population. However, our study’s result showed that mean daily vitamin D intake in the first 4 quintiles in both men and women groups is significantly lower than 10 μg/day, which suggested a serious absence of vitamin D in Vietnamese’s usual meals that may be linked with a high prevalence of VDD in the population. However, participants tend to under-report daily consumption when answering surveys [25, 26], which may lead to a lower result than the actual intake.

As mentioned earlier, over the last few years, vitamin D and its suggested anti-cancer actions have emerged as a hot topic and gained attention from researchers. Studies in vitro and in vivo have shown inhibitory actions upon the binding of Calcitriol to the VDR for many different types of cancers. In their review, Chiang et al. also indicated that the underlying mechanisms of these effects are the VDR-dependent actions, which are mediated through the VDR-dependent transactivation of genomes, which may include anti-proliferative effects via several signaling pathways, inducing apoptosis, anti-angiogenesis, pro-differentiations, anti-inflammation, and DNA repair [27, 28]. The review also nominated non-VDR actions of Calcitriol, as an area of focus in the coming years [27]. Pan et al. reported correlation and interaction among VDR, phosphatase, and tensin homolog deleted on chromosome 10 (PTEN), and epigenetic modifiers such as 5-aza-2-deoxycytidine, trichostatin A (TSA), sodium butyrate upon binding of Calcitriol to VDR may be able to enhance the apoptosis in human GC cells [29]. Also, vitamin D, by altering the expression of genes in the extracellular matrix remodeling, can modify the tumor’s extracellular microenvironment and suppress GC’s progression [30]. Vitamin D may inhibit the expression of numerous Hedgehog signaling target genes, thus decreasing cell viability in GC cell viability [31]. Bao et al. reported that 1,25(OH)2D3 potentiates cisplatin-mediated cell growth inhibition and cell apoptosis in GC cells [32]. *H. pylori* infection is the major risk factor for non-cardia GC [5]. Yang et al. found that sufficient vitamin D levels could be associated with decreased *H. pylori* infection, which may crucially contribute to the prevention of GC [33]. Convincing laboratory evidence at the levels of cells and tissues demonstrated a promising ability of vitamin D in suppressing the tumorigenesis and progression of GC. However, the evidence of epidemiological studies remains paradoxical and inconsistent.

Recently, epidemiologic studies have attempted to prove the relationship between vitamin D status with the incidence and mortality of many types of cancer. However, the findings are conflicting. To evaluate the vitamin D status of participants, measured serum 25OHD (Calcidiol) value was most used. Raimondi et al. reported two most common VDR polymorphisms - FokI and BsmI - reduce the risk of cancer in the breast, skin, and prostate and suggested the same impact on cancer risk at any site in Caucasians [34]. Vaughan-Shaw et al. analyzed 44,165 cases from 64 non-randomized controlled trials (non-RCT) studies, suggesting that a higher serum 25OHD was associated with a 26% lower mortality and a 16% lower rate of progression in cancer patients. However, only one in 64 included studies was related to GC, and this study’s size was small [35]. Giovannucci et al. reached a similar conclusion for total cancers, and a non–statistically significant but suggestive inverse association for GC was recorded [36]. A prospective cohort study from China showed that higher serum 25(OH) D levels had no association with the risk of GC [37]. However, the study model has a relatively low population mean serum 25OHD level, and the mean follow-up time was short (5.25 years). Moreover, after obtaining pre-trial serum 25OHD, they did not collect any follow-up samples. Other non-RCT studies indicated lower serum 25OHD may be a contributory risk for both predisposition and development of GC and associated with poor clinical outcomes [9, 38–40]. The studies assessing vitamin D intake are few. Khayatzadeh et al. analyzed data from 4 case-control studies and found no evidence for the significant association between vitamin D intake as well as serum 25OHD level and risk of GC [41–45]. However, the total study population was small, mainly in Western countries, and the results were inconsistent. Eom Et al investigated 715 pairs of newly diagnosed GC patients and controls in Korea and found no statistically significant interactions between vitamin D intake and risk of GC [46].

Inaccurate assessment of vitamin D status could overstate, or underestimate its relation to tumor development. Although serum 25OHD concentration is a useful biological parameter for assessing current vitamin D status, it constantly varies depending on serum Calcium and Parathyroid hormone (PTH) level, season, and comparability of assays for serum 25OHD [47]. Serum 25OHD measured at a specific moment may not reflect one’s status of vitamin D exposure for a long time. Published data from many studies did not provide time between the measurement of 25OHD and the diagnosis of cancer [48]. Besides, medical treatment and modification in patient’s outdoor sunlight exposure, physical activity, and daily diet after the diagnosis of cancer may reversely result in lower 25OHD levels, thus, a cause-effect association cannot be established [46, 49].

To overcome these issues, some studies, including ours, used estimated vitamin D intake instead. Still, the method also has its weaknesses. The accuracy of estimated intake varies by participants’ abilities to recall types of foods, amount consumed, and frequency of intake. To improve the accuracy, strict follow-up and repeated detailed surveys are desired, despite it will cost vast human resources and financial costs, especially when performing on a large study population in a long observation period. Besides, evaluating the nutrient composition of each type of food and developing a comprehensive survey are also challenging. Also, respondents tend to under-report their consumption, which may lead to a lower result compared to actual intake and the need for precise reassessment [25, 26].

Evidence from epidemiological studies remains controversial, and a causal relationship cannot be built solely from non-RCT study findings. Other micronutrients (e.g., beta carotene, vitamins C and E, folic acid, and selenium) showed their potential in observational studies but failed to prove their anti-cancer effect in RCTs, and some even cause harm at high doses [49]. However, they found no solid data from RCTs to confirm the interrelation between vitamin D status and GC, as GC was the pre-specified outcome. A recently updated meta-analysis of RCTs showed that vitamin D supplementation was associated with 13% reduced total cancer mortality over 3–10 years of follow-up, but not associated with a lower cancer incidence [50]. Still, the heterogeneity in pooled results from types of cancer with dissimilar risk factors was the main weakness. Recent results of the vitamin D status using serum 25-hydroxyvitamin D and gastric cancer, a cross-sectional study in Koreans have confirmed a strong beneficial effect against cancer [38]. A recent review published in 2022 summarized results from observational studies and ten RCTs to review the relation between vitamin D and cancer and showed that RCTs have not been able to confirm findings from observational studies [51]. Two most recent RCTs (in the US in 2019 and in Australia in 2022) showed no beneficial effect of vitamin D supplementation on the incidence or mortality of cancers [52, 53]. However, these studies were conducted on healthy populations with unknown vitamin D deficiency status, and the outcomes were not specific for GC. Furthermore, the intervention duration (5.3 years and 5 years, respectively) and post-intervention follow-up duration (2 years and 5.7 years, respectively) were short. Interestingly, a subgroup analysis of the RCT conducted in the US showed that participants with BMI < 25 kg/m2 had a significantly reduced risk of cancer from vitamin D supplementation [53]. The findings may suggest the beneficial effect of vitamin D against cancer on populations with a low prevalence of obesity, such as the Vietnamese population [54].

The present study has several weaknesses. As discussed above, using an estimated vitamin D intake for the assessment of vitamin D status may not be the optimal method. We did not evaluate other determinants of human vitamin D status such as serum 25OHD concentration, supplements, calcium and PTH levels, skin pigmentation, and sunlight exposure. Despite some limitations, our study suggested low dietary intake of vitamin D may associate with an increased risk of GC in the Vietnamese population. We suggest physicians should assess the vitamin D status of GC patients and consider an appropriate supplement and healthy diet if needed. Nevertheless, further research is needed. We recommend well-designed RCTs with GC as the pre-specified outcome, along with improvements in assessment methods of human vitamin D status, and inclusion of other essential factors such as sunlight exposure. Our study also indicated a low dietary intake of vitamin D in the Vietnamese population, which may lead to a high prevalence of bone-related diseases and perhaps cancer, cardiovascular disease, diabetes, and autoimmune disorders as well [49]. We urge a national plan to improve the nutritional quality of people’s diets.

## Data Availability

The datasets are available from the corresponding author on reasonable request.

## Abbreviations

BMI: Body mass index
GC: Gastric cancer
OR (95%CI): Odds ratio (95% confidence interval)
S.D.: Standard Deviation
SQFFQ: A semi-quantitative food frequency questionnaire
VDD: Vitamin D deficiency
